# Association between social support and mutual-support needs among the rural adults in China: a cross-sectional study

**DOI:** 10.3389/fpubh.2023.1171046

**Published:** 2023-06-02

**Authors:** Xi Tang, Ling Li, Keru Yao, Qin Luo, Lu Zhao, Lu Li, Zhihan Wang, Yijie Fang, Li Huang, Xinhong Yin

**Affiliations:** ^1^School of Nursing, University of South China, Hengyang, China; ^2^Department of Gastroenterology, The Second Affiliated Hospital of University of South China, Hengyang, China; ^3^Health School of Nuclear Industry, Hengyang, China; ^4^Department of Oncology and Hematology, The Second Affiliated Hospital of University of South China, Hengyang, China

**Keywords:** China, mutual-support, adults, rural district, eldercare

## Abstract

**Background:**

In rural China, there is now a huge gap between the supply and demand for old-age care. To close the gap, developing rural mutual old-age services is extremely important. The purpose of this study is to clarify the relationship among social support, mutual support need, and mutual support willingness.

**Methods:**

We conducted an online questionnaire survey using a Chinese Internet research company; 2,102 valid responses were received. The measures comprised the Social Support Rating Scale, the Mutual Support Willingness Questionnaire, and the Mutual Support Needs Scale. We calculated Pearson correlations to explore the association of social support with mutual-support need and mutual-support-need willingness. Multivariate analyses were also conducted using these factors as dependent variables.

**Results:**

The total score for the mutual support need for the adults in rural areas was 58.0 ± 12.1 and 36.96 ± 6.40 for social support, approximately 86.8% of the participants were willing to participate in mutual support. Furthermore, mutual support needs were positively correlated with subjective support (*p* < 0.01) and support utilization (*p* < 0.01), but negatively correlated with willingness to support each other (*p* < 0.05). The need for mutual support was also associated with age, sex, education level, dissatisfaction with the current economic situation, health status, and so on.

**Conclusion:**

It is necessary for government and health care providers to assess the different needs of rural older people and encourage individuals and organizations to provide mutual support for older people, especially to enhance emotional care for older people and improve their use of support. This is of great significance for developing mutual support services in rural China.

## 1. Introduction

Over the past few decades, the population has begun to age rapidly, especially in China. According to data published by the National Health Commission, China entered an ageing society in 2000, and by 2035, the country will have over 400 million people over the age of 60, entering a stage of severe ageing at a rapid rate (1). Among these old people, nearly 60% of the adults population is distributed in rural areas (2). As a result of China’s rapid urbanization, a large number of young people have moved to the cities, leaving an increasing number of adults people in the countryside. In addition to the increasing number of only children in China, the “four-two-one” family model (four older adult people, one couple, and one child) is more common, making it difficult for family members in urban areas to take care of the adults in rural areas. The traditional family care function of relying on children and relatives is weakened. Collectively, it is obvious that relying on family members and professional care is not enough to cater for the growing population of older people in rural areas. For this reason, China draws on the experience of other countries that have entered an aging society earlier to promote the mutual-support model in old age.

The mutual-support adults care service is designed with the help of the pension insurance system; that is, the unhealthy adults are cared for by non-adults people and young, healthy adults people other than family members (3). In the United Kingdom, mutual-support adults care services are primarily provided through “time banking.” Members earn their time by serving others and deposit it in the time bank. When members need help, they draw time from the bank to receive services from others (4). In Germany, the government cooperates with universities to introduce university students to living in the homes of adults widows and orphans and to waive the rent, but the students are expected to help care for the adults (5). The “Villages” model is a membership-based mutual-care community popular in the United States (6). The model provides daily care and spiritual comfort to homebound members through voluntary services in the form of neighborhood mutual support and external service providers (7). The “neighborhood mutual-support network” is a feature of the Japan mutual-support model, which is aimed at people over 65 years of age living in urban communities, especially those who live alone or are widowed. The association organizes various activities such as neighborhood gatherings, fitness, and travel to exchange information, share experiences, relieve loneliness, and enrich themselves among members (8). A study from the US showed that supportive relationships maintain people’s mental health, and the absence of supportive relationships increases the risk of dying from a variety of illnesses (9). This is similar to other studies that have concluded that social support is positively associated with older people’s health, which is more pronounced in developing countries (10, 11). Yoh Murayama found that the mutual support of neighbors and families can be effective in improving the mental health of older people living alone with low economic status (12). Previous studies have shown that such transaction-based mutual support activities can connect social capital and social relationships and have a positive impact on improved physical and mental health and increased social intercourse among older people (13–15).

Previous Chinese studies conducted on the factors influencing the willingness and need for mutual support among the rural adults in China (16, 17). A survey of a poverty-stricken county in Hunan Province showed that the need for health services in mutual support need is highest for poor rural adults (18). A study from rural areas in 12 Chinese provinces shows that the higher the degree of social support, the stronger the willingness to participate in mutual support for the adults (19). Yao Keru (20) held that the rural older Chinese people are strongly influenced by traditional attitudes and are reluctant to leave the familiarity of their place of residence, yet traditional ways of caring are not meeting the needs of older people as family structures change, making them more aware of the importance of support from neighbors and communities.

Consequently, this article focuses on clarifying the association between social support, mutual support willingness, and mutual support needs in rural adults Chinese, particularly when the children do not often accompany. This study is intended to help inform efforts to rationally integrate social resources, develop mutual help services for the adults, and satisfy rural eldercare needs.

## 2. Methods

### 2.1. Participants

The participants were recruited from the residents’ health archive, which is part of China’s basic public health service projects and has a coverage rate of over 90%. We recruited participants from June 2021 to December 2021 in four regions of Hunan Province, China: Changsha, Yueyang, Huaihua, and Hengyang. Participants included those with registered rural residence and those aged 60 and up who live in rural areas.

The electronic questionnaire was promoted and distributed to participants by volunteers from the rural community health centers via WeChat (a free application for instant messaging services on smart terminals). Volunteers provide paper questionnaires to participants who do not have internet access. Participants who are not educated or have difficulty completing the questionnaire on their own may have sought assistance from volunteers or family members. Electronic questionnaire data were collected and stored by Questionstar, a professional online questionnaire, evaluation, and voting website. The study was approved by the Ethics Committee of the University of South China, and all participants signed an informed consent form.

### 2.2. Measurements

#### 2.2.1. Social support

The Social Support Scale (SSRS) was used in this study to assess the degree of social support of individuals. The SSRS consists of 10 items and includes three dimensions: subjective support, objective support, and utilization of social support (21). The subjective support dimension has four items and is social support for the individual’s intrinsic emotional needs. The objective support dimension has three items and is the satisfaction of the individual’s material and physical needs. The degree of support use has three items and depends on how well the individual is able to use social support and feel satisfied in the crisis. For items 1–5 and 8–10 of the scale, the options “none” scored 1, “very little” scored 2, “average” scored 3, and “full support” scored 4 points. For items 6 and 7, if the answer is “no source,” 0 points will be given, and if the answer is “yes source,” several points will be given for each source. As a widely used research tool for measuring social support in China, SSRS has good reliability and validity.

#### 2.2.2. Mutual support willingness

To assess mutual support willingness, participants were asked one question (yes/no), “After understanding mutual care, are you willing to participate in mutual care?”

#### 2.2.3. Mutual support needs

To measure an adults person’s mutual support needs in rural areas, we adopted a scale from a previous study on the mutual pension demand scale among the adults in the community (22). We obtained permission from the authors of the original scale to modify the wording of the items in order to better fit the situation of rural older adults. The modified scale has 18 items and includes 4 dimensions: daily life care needs, health service needs, spiritual comfort needs, and entertainment and learning needs. Participants responded to the stem postulate, “Do you need…” using a 5-point Likert-type scale (5 = always, 4 = often, 3 = sometimes, 2 = rarely, 1 = never). The total score was calculated by summing the item scores. Higher scale scores indicated a greater need for mutual support. The modified Mutual Aging Needs Scale for Older Adults in the Community was retested, and Cronbach’s alpha was used to confirm the reliability of the internal consistency of the instrument (*α* = 0.938). Confirmatory factor analysis (CFA) was used to test the construct validity of the instrument. The one-factor CFA model for the 18-item instrument showed that all fit indices were adequate (root mean square error of approximation = 0.084, comparative fit index = 0.907, goodness of fit index = 0.896, adjusted goodness of fit index = 0.862, and standardized root mean square residual = 0.059).

#### 2.2.4. Demographic characteristics

The general information questionnaire includes factors of gender, marital status, education level, annual family income, child visit frequency, etc. There are 19 items in total. A descriptive analysis of the variables included in this study is shown in Table 1.

**Table 1:** Participants’ characteristics and their associations with mutual support needs (*n* = 2,102).

Independent variable	*n* (%) or mean ± SD (range)	Mutual support need	
		Mean ± SD or Spearman’s *r*	*P*
**Individual characteristic**			
Age (years)			<0.001^a^
60–69	1,243 (59.1)	57.03 ± 12.09	
70–79	626 (29.8)	58.44 ± 11.15	
80~	233 (11.1)	61.72 ± 13.92	
Sex			0.029^b^
Male	899 (42.8)	57.30 ± 12.69	
Female	1,203 (57.2)	58.46 ± 11.66	
Nation			0.771^b^
Other nationality	252 (12.0)	58.17 ± 12.44	
Han nationality	1850 (88.0)	57.94 ± 12.08	
Education level			0.005^a^
Non-educated	375 (17.8)	59.83 ± 12.28	
Primary school	921 (43.8)	57.65 ± 10.95	
Secondary and higher	806 (38.3))	57.46 ± 13.20	
Marital status			0.198^a^
Never married	89 (4.2)	58.61 ± 17.52	
Married	1,528 (72.7)	57.71 ± 11.75	
Divorced	63 (3.0)	56.51 ± 16.64	
Widowed	422 (20.1)	58.98 ± 11.24	
Number of children			0.083^a^
0	42 (2.0)	52.29 ± 18.98	
1	298 (14.2)	58.85 ± 12.75	
2 or more	1762 (83.8)	57.95 ± 11.77	
Child visit frequency			0.296^a^
Never visit	81 (3.9)	54.63 ± 17.66	
Visit occasionally	982 (46.7)	58.27 ± 11.18	
Visit often	738 (35.1)	57.82 ± 12.43	
Live with children	301 (14.3)	58.23 ± 12.43	
Primary caregiver			<0.001^a^
Oneself	707 (33.6)	56.98 ± 12.49	
spouse	938 (44.6)	57.82 ± 11.45	
children	384 (18.3)	60.43 ± 11.79	
Others	73 (3.5)	56.41 ± 16.34	
Relationships among family members			0.039 ^a^
Not harmonious	132 (6.3)	56.47 ± 15.86	
General harmony	482 (22.9)	59.16 ± 12.11	
Harmonious support	1,488 (70.8)	57.71 ± 11.72	
**Economic characteristic**			
Average monthly income			0.427^a^
Less than 2000 yuan	975 (46.4)	58.34 ± 12.15	
200 to 5,000 yuan	454 (21.6)	57.77 ± 10.88	
Over 5,000 yuan	673 (32.0)	57.57 ± 12.85	
Source of income			0.033^c^
Subsistence allowance	279 (13.3)	58.82 ± 11.38	
Wage	457 (21.7)	56.59 ± 11.83	
From children	697 (33.2)	58.38 ± 11.59	
Pension	463 (22.0)	58.59 ± 12.47	
Other	206 (9.8)	57.05 ± 14.31	
Satisfaction with the current economic situation			0.002^a^
No	339 (16.1)	59.41 ± 14.28	
General	956 (45.5)	58.43 ± 10.84	
Yes	807 (38.4)	56.81 ± 12.48	
Main mobile communication equipment			
No			0.003^a^
Basic mobile phones	170 (8.1)	57.88 ± 14.20	
Smartphone	1,120 (53.3)	58.79 ± 11.22	
	812 (38.6)	56.85 ± 12.76	
**Medical characteristic**			
Health condition			<0.001^a^
Unhealthy	392 (18.6)	60.39 ± 13.08	
Suboptimal	889 (42.3)	58.70 ± 11.43	
Healthy	821 (39.1)	56.01 ± 12.09	
Current diseases			<0.001^c^
No disease	731 (34.8)	55.83 ± 12.88	
One or two disease	1,092 (52.0)	58.52 ± 11.27	
Three or more	279 (13.3)	61.41 ± 12.31	
Regular physical examination			0.231^b^
No	1,287 (61.2)	58.22 ± 11.80	
Yes	815 (38.8)	57.57 ± 12.61	
Health knowledge needs			<0.001^a^
No	282 (13.4)	54.80 ± 15.13	
General	934 (44.4)	55.78 ± 10.53	
Yes	886 (42.2)	61.27 ± 11.84	
To the nearest medical facility			0.158^a^
less than 15 min	660 (31.4)	57.21 ± 12.87	
16 to 30 min	628 (29.9)	58.82 ± 10.54	
31 to 45 min	388 (18.5)	57.69 ± 11.53	
46 to 60 min	166 (7.9)	57.96 ± 12.39	
Over 60 min	260 (12.4)	58.24 ± 14.20	
Whether serious illness can be treated in time			<0.001^b^
No	573 (27.3)	60.45 ± 12.90	
Yes	1,529 (72.7)	57.04 ± 11.69	
Mutual-support willingness			<0.001^b^
No	277 (13.2)	68.46 ± 11.62	
Yes	1825 (86.8)	54.70 ± 14.64	

SD, standard deviation.

a Welch’s *t*-test.

b Independent samples *t*-test.

c One-way ANOVA.

### 2.3. Statistical analysis

First, we conducted descriptive analyses of the characteristics of the adults. Second, bivariate analyses between the mutual support needs and other variables were carried out using the one-way analysis of variance (ANOVA), Welch’s *t*-test, and Spearman’s rank correlation coefficients. Lastly, the forced-entry method of multiple regression analysis was used to analyze the factors associated with the mutual support needs. We used the total mutual support needs score variable as the dependent variable. The independent variables were those significantly associated with mutual support needs in the bivariate analyses (including age and sex) (Table 2), social support, and other variables. Except for confirmatory factor analysis, which was carried out using AMOS version 26, all analyses were carried out using IBM SPSS Statistics 21.

**Table 2:** Social support and their associations with mutual support needs (*n* = 2,102).

Independent variable	Mean ± SD	Mutual support needs	
		*r*	*p*
Mutual support needs	57.97 ± 12.10		
Daily life care	14.33 ± 4.32	0.710	<0.01
Health service	14.32 ± 3.18	0.789	<0.01
Spiritual comfort	16.38 ± 3.97	0.870	<0.01
Entertainment and learning	12.93 ± 3.31	0.752	<0.01
Social support	36.96 ± 6.40	0.006	0.786
Objective support	9.07 ± 2.78		
Subjective support	20.32 ± 3.92	0.100	<0.01
Support utilization	7.58 ± 2.02	0.077	<0.01
Mutual-support willingness		−0.092	<0.01

## 3. Results

### 3.1. Characteristics of the participants

Among the 2,167 questionnaires distributed, 65 were excluded because of missing data on items. Ultimately, 2,102 cases were analyzed (total valid response rate: 97%). Table 1 summarizes the participants’ characteristics. 59.1% of the 2,102 study participants were between the ages of 60 and 70, 57.2% were female, and 43.7% could not or only partially care for themselves.

### 3.2. Distribution of responses on mutual support willingness and mutual support needs

Table 1 shows participation in mutual support willingness. Altogether, 1825 (86.8%) of the participants were willing to participate in mutual care after understanding the concept of mutual support; 277 (13.2%) were not willing to participate in mutual support. Table 2 shows the distribution of mutual support needs and social support and their correlations with mutual support needs. The average score on mutual support needs was 57.97 ± 12.10, and the average score on social support was 36.96 ± 6.40. The correlations between mutual support needs and the four mutual support dimensions ranged from 0.71 to 0.87; the highest correlation (0.87) was found for the spiritual comfort dimension. The correlation between mutual support needs and mutual willingness was −0.092; all correlations between mutual support needs and social support were significant, except for objective support.

### 3.3. Factors related to the mutual support need

#### 3.3.1. Bivariate analyses

The association of each independent variable with mutual support needs was examined in a bivariate analysis. The results are shown in Tables 1, 2. Higher scores on the mutual support need score were associated with older age (*p* < 0.001), lower education level (*p* = 0.005), greater dissatisfaction with the current economic situation (*p* < 0.001), poorer health condition (*p* < 0.001), more diseases (*p* < 0.001), and a greater need for health knowledge (*p* < 0.001). The need for support was also associated with sex, the primary caregiver, family relationships, source of income, type of communication device, timely delivery of medical care, and willingness to help each other. The mutual support need was positively related to subjective support (*p* < 0.01) and support utilization (*p* < 0.01) and negatively related to mutual support willingness (*p* < 0.05).

#### 3.3.2. Multivariate analyses

The results of the multiple regression analysis are shown in Table 3. In this model, the adjusted *R*^2^ was 0.166 and the *F*-value was 15.828, with a value of *p* less than 0.001. Mutual support needs (*β* = 0.362, *p* < 0.001) were associated with stronger social support. Mutual support needs were significantly higher for people over 80 years of age than for people in the 60–69 age group (*β* = 3.689, *p* < 0.001), for non-educated people than for people with secondary and higher education (*β* = 1.656, *p* = 0.018), the mutual support need of person to be cared for by someone other than spouses and child was greater than the person to care oneself (*β* = 2.610, *p* < 0.001), the need for mutual support is higher for people with generally harmonious relationships with family members than for those with harmonious relationships (*β* = 1.564, *p* = 0.015), the mutual support need was higher for those whose income relied mainly on pensions than for those who had wage (*β* = 1.598, *p* = 0.041), and the mutual support need was higher for those who considered their current financial situation to be general (*β* = 2.093, *p* < 0.001) and unsatisfied (*β* = 3.412, *p* < 0.001) had higher mutual support need than those who were satisfied, those who did not have a mobile phone had lower mutual support need than those who had an adults phone (*β* = −1.853, *p* = 0.050), and those whose status was general (*β* = 1.605, *p* = 0.010) and unhealthy (*β* = 2.420, *p* = 0.004) had a higher mutual support need compared to those who did not have a disease, those with one or two diseases (*β* = 1.331, *p* = 0.023) and those with a combination of three or more diseases (*β* = 2.698, *p* = 0.002), people with no health knowledge needs (*β* = −5.612, *p* < 0.001) and general health knowledge needs (*β* = −5.332, *p* < 0.001) have higher mutual support needs than those with health knowledge needs, and those who could not seek timely medical care (*β* = 3.476, *p* < 0.001) compared to those who could seek timely medical care. The mutual support need was lower for those who did not have mutual support willingness compared to those who had mutual support willingness (*β* = −3.541, *p* < 0.001).

**Table 3:** Multiple regression results on factors related to mutual support needs (*n* = 2,102).

Variable (reference group)	*β*	*p*
Age (60–69)		
70–79	0.449	0.435
80~	3.689	<0.001
Sex (Man)		
Female	0.636	0.211
Education level (Secondary and higher)		
Non-educated	1.656	0.018
Primary school	0.270	0.636
Primary caregiver (Oneself)		
spouse	0.021	0.970
Others	2.610	<0.001
Relationships among family members (harmony)		
Not harmonious	−0.292	0.797
General harmony	1.564	0.015
Source of income (Wage)		
Subsistence allowance	1.036	0.240
From children	0.953	0.174
Pension	1.598	0.041
Other	0.286	0.765
Satisfaction with the current economic situation (Yes)		
No	3.412	<0.001
General	2.093	<0.001
Main mobile communication equipment (Basic mobile phones)		
No	−1.853	0.050
Smartphone	−0.965	0.090
Health condition (Healthy)		
Unhealthy	2.420	0.004
Suboptimal	1.605	0.010
Current diseases (No disease)		
One or two disease	1.331	0.023
Three or more	2.698	0.002
Health knowledge needs (Yes)		
No	−5.612	<0.001
General	−5.332	<0.001
Whether serious illness can be treated in time (Yes)		
no	3.476	<0.001
Mutual-support willingness (yes)		
no	−3.541	<0.001
Social support	0.362	<0.001
*R*		0.407
*R*^2^		0.166
Adjusted *R*^2^		0.155
*F*		15.828
*P*		<0.001

## 4. Discussion

This study explored important factors in the mutual support needs of rural older adults, with a particular focus on social support. The results showed that subjective support and support utilization in social support were associated with stronger mutual support needs, while objective support in social support did not show a significant association. To our knowledge, few studies have investigated the factors associated with the mutual support need among rural older adults, particularly social support and mutual support willingness. There is a need to understand the mutual support need among rural older adults at the level of social support to develop mutual support services and optimize the allocation of rural retirement resources for healthy aging.

### 4.1. Social support and mutual support need

The results of this study showed that subjective support and support utilization played a substantial role in mutual support needs. In contrast, mutual support needs were not significantly associated with objective support. According to this finding, rural older adults with higher subjective support and support utilization had higher mutual support needs; in short, older people who receive more emotional support and make better use of social support are more likely to engage in mutual support, which is consistent with a study (23) that found that the stronger an older adult’s close and trusting relationships with neighbors, the higher the participation in intergenerational programs (a social service that promotes mutual aid between members of the younger and older generations). Likewise, this result is also reinforced by a study made in Japan (13) and the United Kingdom (24) in which older adults take the initiative in group activities, and positive feedback through atmospheric motivation increases mutual support (25). Regarding willingness to provide mutual support, our study showed that older adults who are willing to help each other have a higher need for mutual help. Some studies suggest that people may be motivated to help others because they need help from others (26, 27). A previous study in poor neighborhoods in The Hague and Utrecht, the Netherlands, also showed that the more residents interact with each other, the more likely they are to share emotions, and the more emotions they share, the more likely they are to build long-term stable neighborhood relationships and become actively engaged in community affairs (28). In rural China, the adults believe that falling leaves settle on the roots. The residents of the villages are acquaintances with similar backgrounds and can better understand each other’s needs (29, 30). This may also be the reason why adults people are willing to live out their lives in villages where acquaintances take care of each other.

### 4.2. Other factors related to the mutual support need

This study also revealed that medical, economic, and sociodemographic factors, including age, sex, education level, primary caregiver, primary source of income, satisfaction with the economy, means of communication, health status, number of illnesses, health knowledge needs, and timely access to medical care, were the main predictors of the mutual support need among rural adults. Specifically, this study showed that the mutual support need was higher for those aged 80 years and older compared to those aged 60–69 years, and the mutual support need was higher for women compared to men. Similarly, the mutual support need was higher among the adults who attended secondary and higher education, respectively, compared to non-educated ones. Previous studies (29, 31, 32) have suggested that adults people in rural areas need more outside support due to lower education levels and children moving to cities, so they lack care and have less health information skills.

The need for mutual support is higher for the adults who are cared for by others (non-spouse children) compared to self-care. This study also shows that the primary caregivers of rural older adults are themselves or their spouses. As in China, many Asian countries are influenced by Confucianism, which upholds “filial piety,” reflecting young people’s duties to care for older people within the family unit, so that caregivers of older adults are usually their adult children and/or extended family members in developing countries (33, 34). Due to China’s large population base, there are also a relatively large number of adults people living alone, especially in rural areas (35). Without children or family members to care for them, these families will have to rely on society to provide additional services to support them. A survey in Uganda shows that family-based traditions of care for older people are coming under pressure due to local socio-economic transitions, the exodus of youth from rural areas, and the death of a proportion of young people due to HIV infection (36). The tradition of family-based care is being challenged in many developing countries undergoing economic transition, where the need for mutual aid in old age is higher.

Regarding financial status, the mutual support need is higher for the adults whose income is mainly derived from pensions compared to those whose income is mainly derived from wages. In contrast, the adults who were satisfied with their current economic status had a lower mutual support need. This is consistent with a study conducted in an urban village in Guangzhou, China, where higher household income facilitated respondents’ access to neighbors’ help and thus influenced neighborhood mutual support (37). In addition, the mutual support need is higher among older people without mobile phones than among those with mobile phones. A survey conducted by Peter (38) found that health apps using cell phones can help mutual support groups treat addictive behaviors, and some studies have used the “Internet + smartphones model” to meet the aging needs of older adults in the community (39, 40). These findings suggest that it is important that older adults with cell phones, especially those with smartphones, can meet their mutual support needs through the software and information on their devices.

Regarding medical care, the mutual support need was higher among older adults with multiple diseases and unhealthy conditions compared to those who had no diseases and were consciously healthy. The mutual support need was higher among older adults who were unable to seek medical care in a timely manner than those who were able to do so. Previous study has suggested that the level of emotional disorders and alcohol addiction is associated with mutual support (41, 42). This result thus implies that poorer health leads to seeking help from others, which may lead to a stronger need for mutual support. Likewise, a scoping review found that people with mental illness and distressing experiences can be helped through peer or mutual support (43). A survey with people from different regions and household income brackets in Brazil conducted by Petra (44) found that social solidarity can be fostered to reduce harm from COVID-19 through mutual support at the core of the family, apartment, and community. The results of these studies suggested that mutual support can improve people’s mental health and help them better cope with public health emergencies.

## 5. Conclusion

This research showed that subjective support and support utilization were associated with mutual support needs. Additionally, medical and socio-demographic factors, which include age, sex, education level, primary caregiver, family relationships, household income, financial satisfaction, mobile communication equipment, health condition, health knowledge needs, and type of illness, were substantial factors in the need for mutual support for older people in rural areas. Likewise, meeting the mutual support needs for rural older adults has a positive impact on improving their physical and mental health. In rural China, there is now a major imbalance between supply and demand for adults care. To close the gap, developing rural mutual old-age services is extremely important. The government should increase funding for rural mutual support and encourage the community and the surplus rural population to join volunteer activities. There is also a need to assess the support needs of the adults, the uneducated, empty nesters, those without access to the media, and those living far from health facilities and to develop care service plans to improve support utilization. Health care providers should participate in mutual support activities to expand the range of health care services and provide regular emotional care, medical care, medical check-ups, and home visits to key populations.

## 6. Limitations and future directions

This study has several limitations, First, it was conducted in one district in central China, and the sample may have limitations, but Hunan Province, as an agricultural province in China, has typical characteristics of the coexistence of a low-young and adults population in rural China (45), which can reflect the current situation in rural China to some extent. Secondly, this study relied on cross-sectional data, and, therefore, we cannot assess causality. More studies are needed to better understand the mechanisms of associations between social support and mutual-support willingness and need among the rural adults in China.

## Data availability statement

The original contributions presented in the study are included in the article/supplementary material, further inquiries can be directed to the corresponding authors.

## Ethics statement

The studies involving human participants were reviewed and approved by the Ethic Committee of University of South China. The patients/participants provided their written informed consent to participate in this study.

## Author contributions

XT and LiL wrote the main content of this manuscript. XY and LH directed the research framework and questions. KY, QL, and YF participated in the data collection and analysis. LZ, LuL, and ZW contributed to the interpretation of the paper. All authors contributed to the article and approved the submitted version.

## Funding

The study was supported by the Hunan Social Science Foundation (Grant No. XSP20ZDI007), Scientific Research Project of Hunan Provincial Education Department (Grant No. 22C0215), Teaching Reform Project of Hunan Province General Higher Education Institution (Grant No. [2019]457), Natural Science Foundation of Hunan Province (Grant No. 2023JJ30528), Special Science Popularization Project of Hunan innovative Province Construction (Grant No. 2022ZK4291), University of South China Research Projects (Grant No. nk2020209).

## Conflict of interest

The authors declare that the research was conducted in the absence of any commercial or financial relationships that could be construed as a potential conflict of interest.

## Publisher’s note

All claims expressed in this article are solely those of the authors and do not necessarily represent those of their affiliated organizations, or those of the publisher, the editors and the reviewers. Any product that may be evaluated in this article, or claim that may be made by its manufacturer, is not guaranteed or endorsed by the publisher.
